# Anthelmintic-Like Activity and Ultrastructure Changes Produced by Two Polyphenolic Combinations against *Cooperia punctata* Adult Worms and Infective Larvae

**DOI:** 10.3390/pathogens12050744

**Published:** 2023-05-22

**Authors:** Elke von Son-de Fernex, Estefanía Zúñiga-Olivos, Luis Felipe Jiménez-García, Pedro Mendoza-de Gives

**Affiliations:** 1 Teaching, Research and Extension in Tropical Livestock Center, Faculty of Veterinary Medicine and Zootechnics, National Autonomous University of Mexico, Martínez de la Torre, Veracruz 93600, Mexico; 2 Department of Cellular Biology of the Sciences Faculty, National Autonomous University of Mexico, Av. Universidad 3000, Circuito Exterior s/n Alcaldía Coyoacán, Ciudad Universitaria, Ciudad de México 04510, Mexico; 3 Laboratory of Helminthology, National Centre for Disciplinary Research in Animal Health and Innocuity (CENID-SAI), National Institute for Research in Forestry, Agriculture and Livestock, INIFAP-SADER, Jiutepec 62574, Mexico

**Keywords:** nematode motility, SEM, TEM, coumarin, caffeic acid, quercetin, rutin, phytochemicals

## Abstract

*Cooperia punctata* is one of the most prevalent gastrointestinal nematodes affecting cattle under grazing conditions, and the increasing reports of anthelmintic resistance forces researchers to look for novel control measures. Previous reports have proposed the use of polyphenolic compound (PC) combinations (Coumarin:Quercetin (CuQ) and Caffeic-acid:Rutin (CaR)) against free-living stages (L_3_) of *C. punctata*. The objective of this study was to assess the in vitro motility inhibition of *C. punctata* adult worms and infective larvae using the Larval Motility Inhibition Assay (LMIA) and Adult Motility Inhibition Assay (AMIA), and to assess the structural and ultrastructural changes induced by both treatments using Scanning and Transmission Electron Microscopy. For the LMIA, infective larvae were incubated for 3 h in 0.8 mg mL^−1^ and 0.84 mg mL^−1^ of CuQ and CaR, respectively. For AMIA, six concentrations and five incubation periods (2, 4, 6, 12 and 24 h) were assessed using each PC combination. *Cooperia punctata* motility was calculated as a percentage and corrected using control motility percentages. A multiple comparisons Brown–Forsythe and Welch ANOVA test was used to compare larval motility; and to fit the dose–response in AMIA, data were analyzed with a non-linear regression four-parameter logistic equation with a variable slope, using the computer program GraphPad Prism^®^ V.9.2.0. Although larval motility was barely affected by both treatments (*p* > 0.05), adult worm motility was inhibited 100% and 86.9% after 24 h incubation with CuQ and CaR, respectively (*p* < 0.05). The best fit EC_50_ for adult worm motility inhibition were 0.073 ± 0.071 mg mL^−1^ and 0.051 ± 0.164 mg mL^−1^ for CuQ and CaR, respectively. Main structural and ultrastructural lesions observed in both biological stages were: (i) L_3_ sheath–cuticle complex disruption, (ii) collagen fibers degradation; (iii) hypodermic detachment, (iv) seam cell apoptosis and (v) mitochondrial swelling. The alterations observed suggest that the PC combinations interfere with the anatomy and physiology of the locomotive apparatus of the nematodes.

## 1. Introduction

*Cooperia punctata* represents one of the most prevalent and pathogenic species among gastrointestinal nematodes (GIN) affecting grazing cattle in the tropics [1]. Gastrointestinal nematodes are one of the weakest links of animal health in grazing cattle industry; not only due to their negative impact upon health, welfare, and producer’s economy, but also due to the emergence of anthelmintic resistance and the challenge it represents to establish effective control strategies [1]. Polyphenolic compounds (PCs) are one of the largest and most bioactive groups of secondary metabolites of plants (SMP), and for the past decade PCs have been targeted for drug development research due to their highly antioxidant activity, which has been considered a key step for their broad bioactivity spectrum [2,3]. It has been proposed that one of the possible reasons restricting the use of bioactive plants for GIN control might be the low yield of bioactive SMP obtained through organic extraction [4]; however, bioactive molecules obtained and identified through bio-guided fractionation procedures could be assessed for anthelmintic (AH)-like drug development. Most screening of novel compound assays use eggs and larval stages of the parasites due to the challenges and costs that assessing adult worms represent [5]. However, in vitro screening of molecules with living adult worms is the ultimate target [6]. Recently, Escareño et al. (2019) [7] reported the AH-like activity of five commercial PCs (coumarin, rutin, quercetin and caffeic acid) and their interactions against free-living stages of the cattle nematode *Cooperia punctata*. However, the combinations reported as bioactive have not been assessed against adult worms, and little is known regarding the possible mechanisms involved in the bioactivity. Thus, the objective of this study was to assess the inhibitory activity of two PC combinations (coumarin: quercetin and caffeic acid: rutin) on the motility of both infective larvae and adult worms of *C. punctata*, and the structural and ultrastructure changes produced by PC combinations.

## 2. Materials and Methods

### 2.1. Allocation

This study was performed at the Teaching, Research and Extension in Tropical Livestock Center, Faculty of Veterinary Medicine and Zootechnics, National Autonomous University of Mexico, Martínez de la Torre, Veracruz, México.

### 2.2. Polyphenolic Compounds and Combinations

Quercetin (C_15_H_10_O_7_·2H_2_O), Caffeic acid (C_9_H_8_O_4_), Rutin (C_27_H_30_O_16_·xH_2_O) and Coumarin (C_9_H_6_O_2_), with Chemical Abstracts Service (CAS) registry numbers^®^ 117-39-5, 331-39-5, 153-18-4, and 91-64-5, respectively, were obtained from Sigma (St. Louis, MO, USA). As proposed by Escareño et al. (2019) [7], combinations were performed by mixing Coumarin with Quercetin (CuQ) and Caffeic acid with Rutin (CaR) in an 8:2 ratio, as it was the ratio obtained through HPLC in the bioactive fraction of the plant extract [8].

### 2.3. Biological Material

#### 2.3.1. Infective Larvae

*Cooperia punctata* infective larvae (L_3_) were obtained from a donor calf with a mono-specific infection of *C. punctata* (isolate *C*. *p*. CEIEGT-FMVZ-UNAM strain, Mexico [9]). Biological material recovery and calf housing were performed as described by Escareño et al. (2019) [7]. At all times, calf management complied with the Internal Committee for Care and Use of Experimental Animals of the National Autonomous University of Mexico regulations [CICUA-UNAM No. 642].

#### 2.3.2. Adult Worm Recovery

One helminth free calf (raised indoors since birth and treated 10 days prior to infection with albendazole at a dose of 7.5 mg/kg live weight) was orally infected with 84,400 third stage larvae (L_3_) of *C. punctata* (isolate *C*. *p*. CEIEGT-FMVZ-UNAM strain, Mexico; [9]). The calf was kept indoors on straw and was fed with hay, pellets, and milk. The infected calf was slaughtered six days after reaching the patent period by bleeding through opening the carotid artery immediately following captive bolt stunning (in strict accordance with the Internal Committee for the Care and Use of Experimental Animals of the National Autonomous University of Mexico regulations [CICUA-UNAM No. 642]). Worms were recovered from the small intestine following migration through a 250 μm sieve and into a glass container with physiological saline solution (0.9% NaCl) previously heated and kept at 37 °C. Adult worms were then manually recovered, washed three times with pre-heated physiological saline solution (37 °C), and individually examined using a stereomicroscope to confirm their integrity. Right after being examined, worms were incubated in the different PC combinations (see Section 2.4.2).

### 2.4. Evaluation of Polyphenolic Compounds Combinations (PCC)

#### 2.4.1. Larval Motility Inhibition Assay (LMIA)

The LMIA was used to evaluate the effect of each polyphenolic compound combination on the mobility of four-weeks-old ensheathed *C*. *punctata* L_3_ [10]. A thousand ensheathed L_3_ mL^−1^ were placed in 15 mL falcon tubes and incubated on each combination to be tested (0.8 mg mL^−1^ and 0.84 mg mL^−1^ of CuQ and CaR, respectively) and in ethanol at 2.5% (negative control). Thiabendazole (TBZ) 99% (Sigma^®^, St. Louis, MO, USA) was used as positive control at a concentration of 10 mg/mL^−1^. All incubations were carried out for 3 h at 21.3 °C. Afterwards, L_3_ from each tube were immediately washed with distilled water and centrifuged (908× *g*) three times. Larvae were then transferred to sieves (inserts equipped with a 20 μm mesh positioned in a conical tube). After 3 h at room temperature (26 °C), the number of L_3_ migrated through the mesh were counted. The percentage of migration was calculated as %M = (M/T) × 100 (where T represents the total number of L_3_ deposited on the sieve and M stands for the number of L_3_ that had migrated). Four replicates were run for each combination and control.

#### 2.4.2. Adult Motility Inhibition Assay (AMIA)

Immediately after recovery, 13–15 motile *C*. *punctata* adult males were gently washed three times in a physiological saline solution (PSS 0.09%, Pisa^®^ JAL, MX) previously heated at 37 °C, and placed in a 6-well cell culture cluster, with their corresponding treatment. Polyphenolic combinations proposed by Escareño et al. (2019) [7]: Coumarin:Quercetin (CuQ) and Caffeic acid:Rutin (CaR) in an 8:2 ratio were used to obtain the mean effective concentration (EC_50_) against adult worms. A total of 3920 ± 10.4 (Mean ± SE) *C*. *punctata* adult worms of were used to assess the effect of the PCs-combinations on *C*. *punctata* adult motility over the time (2, 4, 6, 12 and 24 h). For the CuQ combination, *C*. *punctata* adult worms were incubated in multiple 6-well cell culture clusters at increasing concentrations of 0.022, 0.043, 0.087, 0.175, 0.35, and 0.8 mg/mL^−1^ of physiological saline solution (PSS 0.09%, Pisa^®^). For the CaR combination, adult worms were incubated at increasing concentrations of 0.026, 0.052, 0.105, 0.21, 0.42, and 0.84 mg/mL^−1^ of physiological saline solution (PSS 0.09%, Pisa^®^). Wells containing 2.5% ethanol diluted in physiological saline solution (0.09% NaCl, Pisa^®^) were used as negative control. Cell culture clusters were incubated in a CO_2_ incubator (37 °C with a 5% CO_2_ inclusion) (CO_2_-Incubator C-150 Binder^®^). Four replicates were run for each concentration, time lapse and control. Worm motility was examined at 2, 4, 6, 12 and 24 h after incubation using an inverted microscope (Nikon Eclipse Ts2^®^), and the number of motile and non-motile *C*. *punctata* (no movement detected in 10 s) was recorded [11]. Individual adult motility per well was calculated as: Worm motility % = 100 × (number of motile worms in the concentration/total number of worms in the same concentration). Finally, motility was adjusted using control motility percentages. Worm motility percentages correction per concentration was calculated using the following formula: Adult motility corrected % (AMc) = 100 − (100 × (1 − MwTx/MwC)). Where MwTX represents the motility percentage of worms incubated in the treatment, and MwC represents the motility percentage of worms in the control group.

#### 2.4.3. Polyphenolic Combinations Effect over the Ultrastructure of *C. punctata* Infective Larvae and Adult Stages

Approximately 1000 ensheathed *C*. *punctata* L_3_ incubated for 3 h in 0.8 mg mL^−1^ and 0.84 mg mL^−1^ of CuQ and CaR, respectively; and 30 adult worms incubated for 24 h in the concentrations 0.08 mg mL^−1^ and 0.052 mg mL^−1^ of CuQ and CaR, respectively; were used to assess structural and ultrastructure changes in both *C*. *punctata* stages. For the control groups L_3_ were incubated in 2.5% ethanol diluted in distilled water and adult worms in 2.5% ethanol diluted in physiological saline solution (0.09% NaCl, Pisa^®^). Four replicates were assessed for each combination and control. After incubation, L_3_ were washed and centrifuged (908× *g*) three times in DW (pH 7.2), placed in the fixing solution (see Section 2.5) and stored at 4 °C. Adult worms were individually washed in physiological saline solution (0.09% NaCl, Pisa^®^), fixed and stored at 4 °C until processed for ultrastructure analysis (see Section 2.5).

### 2.5. Scanning and Transmission Electron Microscopy (TEM and SEM)

Immediately after incubation (see Section 2.4.1 and 2.4.2) *C. punctata* L_3_ and adult worms (12,000 and 360, respectively) were placed in 15 mL falcon tubes, fixated with paraformaldehyde, and transported at 4 °C for further processing [12]. Scanning and Transmission Electron Microscopy samples were processed at the Electron Microscopy laboratory of the biology area and cellular biology department, respectively, from the Science Faculty UNAM, Mexico. At the laboratory, samples were washed with PBS to remove the fixer, followed by a second fixation with osmium tetroxide 2%; L_3_ were fixated for 1 h while adult worms were kept overnight. After fixation, samples were dehydrated using increasing ethanol series [13]. Finally, samples were equally divided for further SEM and TEM processing.

Samples for SEM (6000 L_3_ and 180 adult worms) were dried with CO_2_ in a critical-point dryer (BAL-TEC, CPD 030^®^) and coated with gold for 5 min at an ionizer (DESK II DENTON, VACUUM^®^). Finally, both L_3_ and adult worms were observed under a Scanning Electron Microscope (JSM-5310LV, JEOL^®^ TKY, JP) at an accelerating voltage of 15 and 20 kV.

Samples destinated for TEM (6000 L_3_ and 180 adult worms) were pre-embedded with propylene’s oxide and Epon 812 resin (1:1) for 24 h; afterwards, samples were embedded in polymerized epoxy resin at 60 °C for 24 h. Semi-thin sections (250 nm) were performed and stained with toluidine blue to obtain a general description under an optic microscope. Ultra-thin sections (60 nm) were made using a microtome (Ultracut R, Leica^®^ WETZLAR, DE) and samples were placed on formvar-coated cooper grids. Afterwards, samples were contrasted first with uranyl acetate 4% (20 min and 30 min for L_3_ and adult worms, respectively) and then with lead citrate 0.3% for 10 min [9]. Finally, observation of the ultra-thin sections was performed using a Transmission Electron Microscope (JEM 1010, JEOL^®^) operating at 80 kV. The Digital Micrograph V.2.32.888.0 software was used to obtain the microphotographs.

The alterations observed in the specimens were counted and expressed as percentages. Percentages were obtained after being corrected with the control group using the following formula: Percentage = ((100 − nC)/N) × nTx. Where nC stands for the number of specimens presenting the alteration, N is the total number of specimens assessed, and nTx stands for the number of specimens presenting the alteration in the treated group.

### 2.6. Statistical Analyses

A multiple comparisons Brown–Forsythe and Welch ANOVA test was used to compare larval motility (GraphPad Prism^®^, LLC V.9.2.0.). To fit the dose–response in AMIA, data were analyzed with a non-linear regression four-parameter logistic equation with a variable slope, using the computer program GraphPad Prism^®^ V.9.2.0. All analyses were performed after transforming the data into logarithms (X = logX) and constraining the bottom and top values to 0 and 100, respectively. Finally, EC_50_, 95% confidence limits and R^2^ values were also calculated.

## 3. Results

### 3.1. Effect of Polyphenolic Combinations on the Motility of C. punctata Infective Larvae

Larval migration in the negative control was 86.44 ± 3.22%; while the positive control (Thiabendazole) migrated 9.02 ± 5.42% of *C*. *punctata* larval motility. Both PC combinations showed a low motility inhibition (*p* > 0.05); thus, CaR had a higher inhibitory activity (13.95 ± 4.84) than the CuQ combination (Table 1; *p* < 0.05).

### 3.2. Effect of PCs-Combinations on the Motility of C. punctata Adult Worms

**Figure 1:**
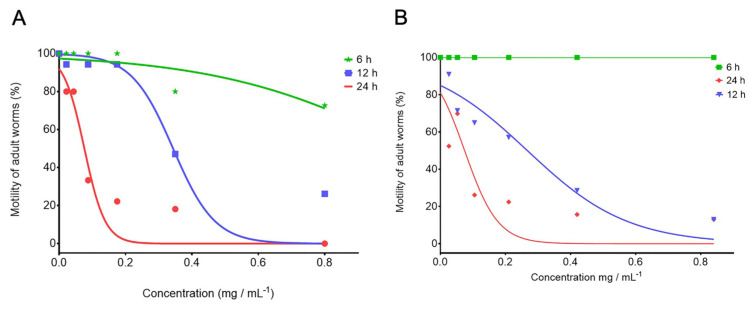
Effect of increasing concentrations of the coumarin:quercetin and caffeic-acid:rutin combinations over the motility of *Cooperia punctata* adult worms at different time lapse (6, 12 and 24 h). (**A**) Motility inhibition of adult worms incubated in CuQ; (**B**) motility inhibition of adult worms incubated in CaR.

Adult worms from the negative control showed a motility of 100 ± 0.00%, 83.41 ± 1.59% and 74.54 ± 6.17% at 6, 12 and 24 h post-treatment, respectively. The motility of adult worms using both PC combinations was not affected after 2 h, 4 h and 6 h post-exposure to the CaR; however, after a 6 h incubation with CuQ, the motility was inhibited in 27.3%. The adult motility at 12 and 24 h after incubation in CaR was 87.01% and 86.9%, respectively; conversely to the inhibition observed with CuQ, where at the maximal concentration, motility inhibition reached 73.81% and 100% after 12 and 24 h, respectively. The best-fit EC_50_ showing a higher coefficient of determination (R^2^) was CuQ after 24 h (0.073 ± 0.071 mg mL^−1^; R^2^ 0.94) and CaR after 12 h exposure (0.192 ± 0.061 mg mL^−1^; R^2^ 0.95) (Figure 1). Mean effective concentrations, 95% confidence intervals and R^2^ are presented in Table 2.

### 3.3. Scanning Electron Microscopy (SEM)

**Figure 2:**
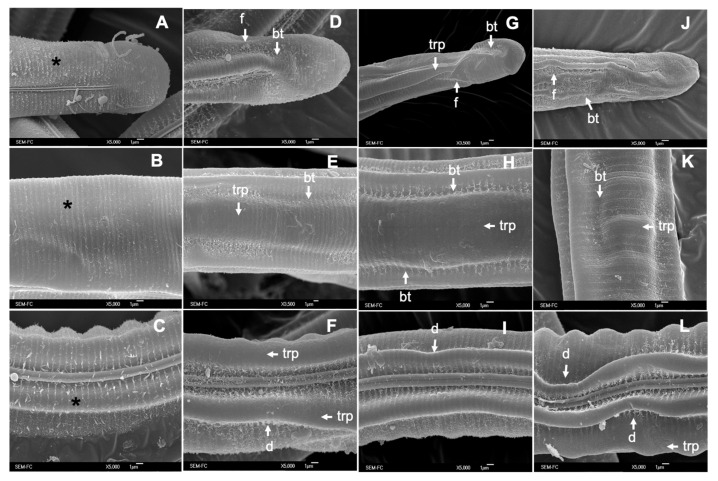
Scanning Electron Microscopy of *Cooperia punctata* L_3_. (**A**–**C**) Infective larvae incubated in ethanol 2.5% with no evident alterations of the sheath apart from a slight loss of turgor (control group; black asterisk); (**D**–**F**) infective larvae incubated in CuQ with evident furrows (f) and depressions (d) on the sheath; (**G**–**I**) infective larvae incubated in CaR with a marked loss of body turgor (bt) and loss of definition in the transversal ridges patter (trp); and (**J**–**L**) infective larvae incubated in TBZ with loss of body turgor and cylindric shape.

The 80% of infective larvae analyzed from the control group (ethanol 2.5%) maintained the structural integrity of the sheath with a normal transversal and longitudinal ridges pattern (black asterisk; trp). The cylindric shape of the body was kept in 80% of the L_3_, however, a slight loss of turgor observed in all specimens (Figure 2A–C). Conversely, 96% of *C*. *punctata* L_3_ exposed to PCs-combinations and thiabendazole evidenced a loss of structural integrity of the sheath (Figure 2D–L). In a generalized manner, 77.4% and 88.2% of the L_3_ incubated in CuQ and CaR, respectively, lost their cylindric shape and presented an irregular surface of both the cephalic and body regions (Figure 2D,G). Furthermore, over 80% of the L_3_ incubated both PC combinations and TBZ showed a shrunken appearance with a loss of body turgor (bt) and multiple furrows (f) and depressions (d) along the sheath, and a loss of definition of the transversal ridges pattern in a diffuse focal pattern (trp).

**Figure 3:**
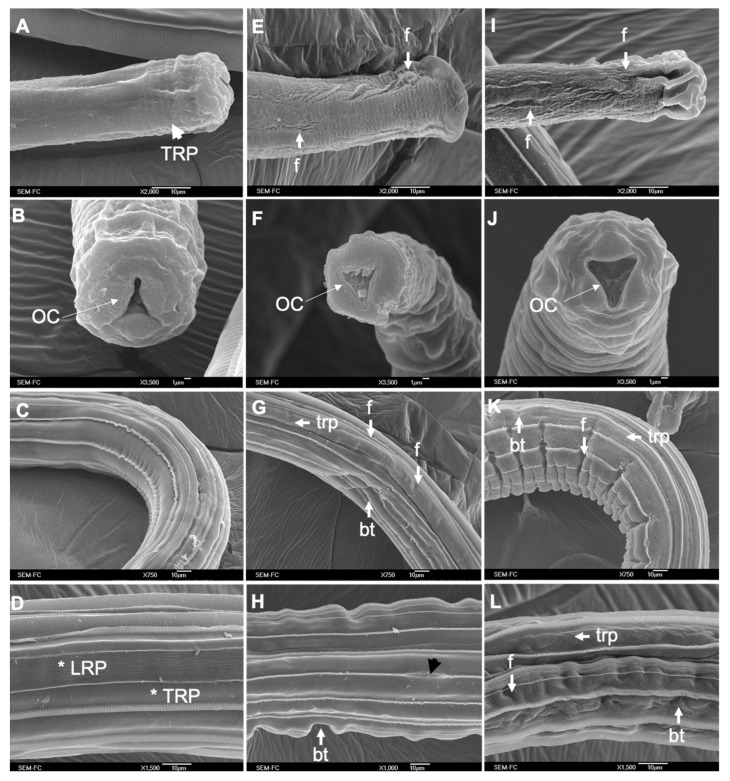
Scanning Electron Microscopy of *Cooperia punctata* adult worms. (**A**–**D**) Adult worms incubated in ethanol 2.5% (control group); (**E**–**H**) adult worms incubated in CuQ with visible loss of body turgor (bt) and cylindrical shape, with multiple furrows (f) along the cuticle, loss of definition and partial deformation of the longitudinal ridges (black arrow); (**I**–**L**) adult worms incubated in CaR showing a collapsed cephalic region, loss of bt and trp.

Scanning Electron Microscopy of adult worms showed that 86.67% of the specimens incubated for 24 h in ethanol 2.5% had a regular cylindric shape with a slight loss of body turgor (bt) and with a normal pattern of both longitudinal and transverse ridges (LRP and TRP, respectively) (negative control; Figure 3A–D). However, 89.18% to 91% of *C*. *punctata* worms exposed to the PCs-combinations had an increased number of cuticular furrows (f) and depressions (d); a marked loss of body turgor (bt) giving the appearance of cuticular stiffness, and a loss of the typical cylindric shape of nematodes (Figure 3D–L). Furthermore, over 90% of the adult worms exposed to the PCs-combinations showed a loss of the transversal ridges pattern definition (trp) and partial deformation of the longitudinal ridges pattern (Figure 3H; black arrow).

### 3.4. Transmission Electron Microscopy (TEM)

#### 3.4.1. *Cooperia punctata* Infective Larvae L_3_

**Figure 4:**
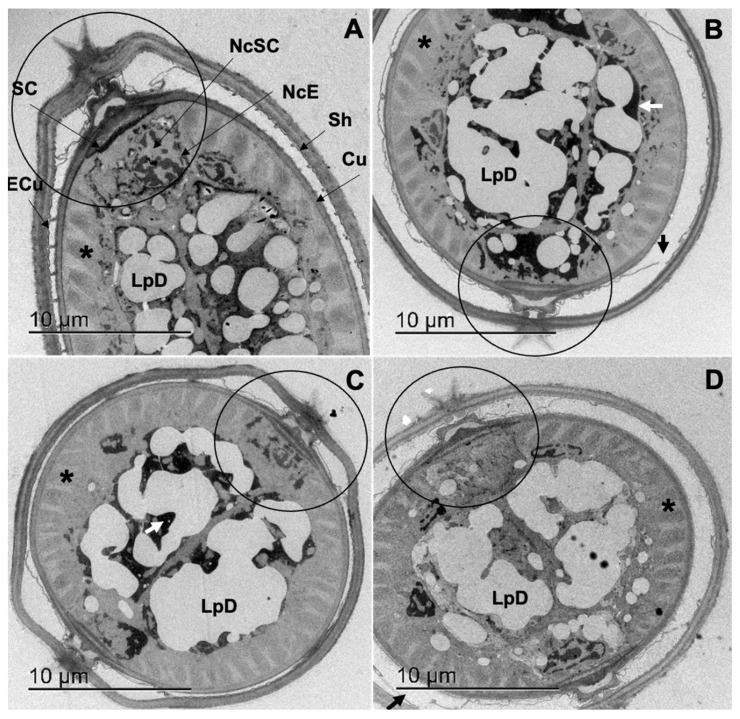
Transversal cuts of *Cooperia punctata* infective larvae observed through Transmission Electron Microscopy. (**A**) Infective larvae incubated in ethanol 2.5% (control group); (**B**) infective larvae incubated in CuQ with a loss of epicuticle continuity and electron-densification of the seam cell and intestinal cells cytoplasm; (**C**) infective larvae incubated in CaR show marked alterations in the seam cell and lateral ending of the L3 body; (**D**) infective larvae incubated in TBZ with epicuticle and sarcomeres alterations.

Figure 4A–D shows the cuticle-sheath complex and internal ultrastructure of *C*. *punctata* L_3_ incubated in the control group (ethanol 2.5%), CuQ, CaR and TBZ, respectively. In the control group, only 26.67% of the L_3_ presented a detachment of the sheath, while 96% of the specimens incubated in CuQ and TBZ groups and 57.6% of the L_3_ incubated in CaR displayed sheath detachment or epicuticle loss of continuity (Figure 4B,D; black arrow), and a slight attenuation of the sarcomeres electron-density (Figure 4A–D; black asterisk).

**Figure 5:**
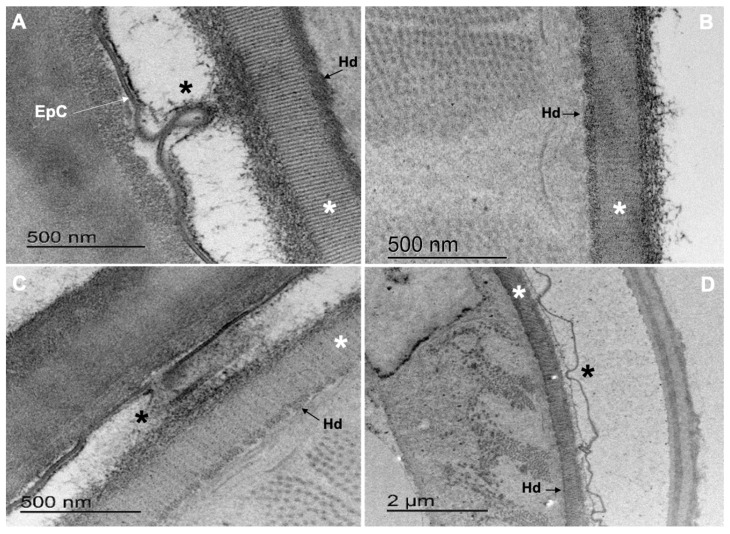
Sheath–cuticle complex of *Cooperia punctata* infective larvae observed under Transmission Electron Microscopy. (**A**) Epicuticle (EpC) and hemidesmosomes (Hd) from infective larvae incubated in ethanol 2.5% (control group); (**B**) infective larvae incubated in CuQ with marked degeneration of collagen bands from the basal zone (BZ; white asterisk) and hemidesmosomes (Hd); (**C**) infective larvae incubated in CaR with loss of EpC continuity and marked degeneration of collagen bands from the basal zone (BZ; white asterisk) and Hd; (**D**) infective larvae incubated in TBZ showing severe alterations of the epicuticle (black asterisk), and degeneration of sarcomeres.

**Figure 6:**
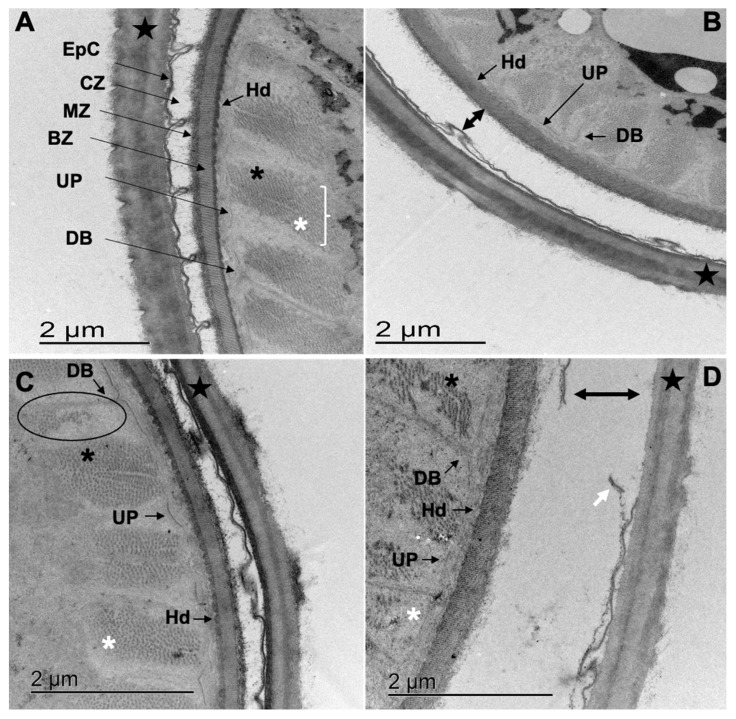
Cuticle and sarcomeres of *Cooperia punctata* infective larvae observed under Transmission Electron Microscopy. (**A**) Infective larvae incubated in ethanol 2.5% (control group); (**B**) infective larvae incubated in CuQ with and increased space in the cortical zone (CZ) and loss of Hemidesmosomes (Hd) definition; (**C**) infective larvae incubated in CaR show alterations in the EpC, sarcomeres, dense bodies (DB), union plates (UP) and hemidesmosomes (Hd); (**D**) infective larvae incubated in TBZ with noticeable alterations of the EpC, DB, UP, H and strong degeneration of sarcomeres.

The EpC of treated larvae presented a different degree of breakage (Figure 5C,D; Figure 6D, double headed arrow and white arrow), a loss of furrows and union points that hold together the sheath and the cuticle (black asterisks, Figure 5C,D). Enlargement of the cortical zone associated to the detachment of the EpC from the MZ (Figure 6B,D) causing the loss of the characteristic pseudometric annulations was also observed (Figure 6B–D). Slimming and loss of the epicuticle integrity combined with the loss of the union points of the sheath and cuticle (black asterisk) were observed in 26.67%, 96%, 57.6% and 96% of the L_3_ incubated in ethanol 2.5% (negative control), CuQ, CaR and TBZ, respectively (Figure 5C,D; Figure 6B–D).

Furthermore, with both PC combinations, treated L_3_ also presented a generalized electron densification of the intestinal cells cytoplasm (Figure 4B,C; white arrow) and an evident coalescence of the lipidic droplets (LpD; Figure 4B–D).

**Figure 7:**
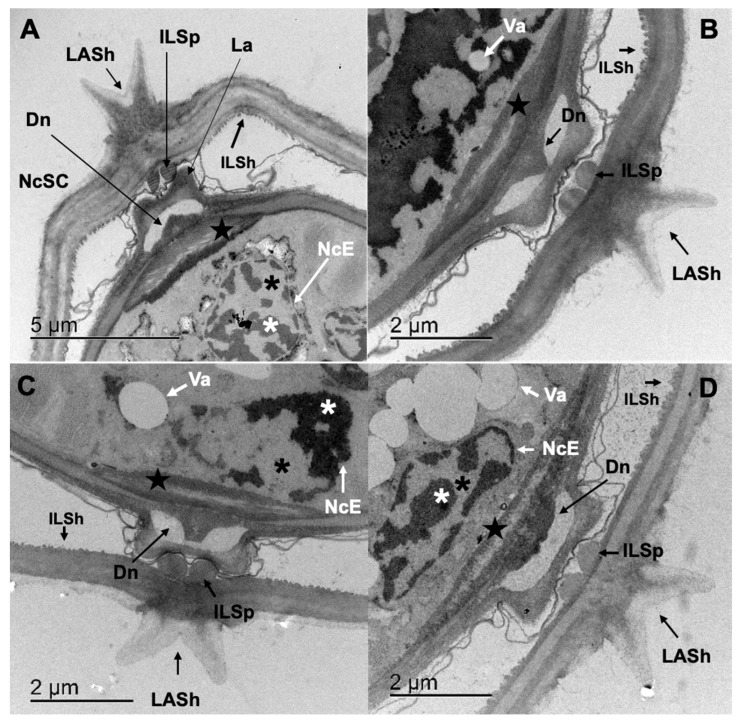
Lateral end of *Cooperia punctata* infective larvae observed through Transmission Electron Microscopy. (**A**) Infective larvae incubated in ethanol 2.5% (control group) where structures and ultrastructures such as lateral alae of the sheath (LASh), internal layer of the sheath (ILSh), internal layer of the sheath specializations (ILSp), lateral alae (La), deirid-like neuron (Dn), deirid-like neuron base (black star), seam cell nucleous (NcSC), seam cell nuclear envelope (NcE), euchromatin (black asterisk), heterochromatin (white asterisk) and internal layer of the sheath (ILSh) can be observed; (**B**,**C**) infective larvae incubated in CuQ and CaR, respectively. With a noticeable loss of the transverse band pattern of the ILSp and the cilia of the deirid-like neuron tip (Dn) and deirid-like neuron base. Loss of the nuclear envelope (NcE) and condensation and margination of the heterochromatin within the nucleus; (**D**) infective larvae incubated in TBZ presenting the same alterations previously described and a high degree of vacuolization (Va) in the nucleus cytoplasm.

**Figure 8:**
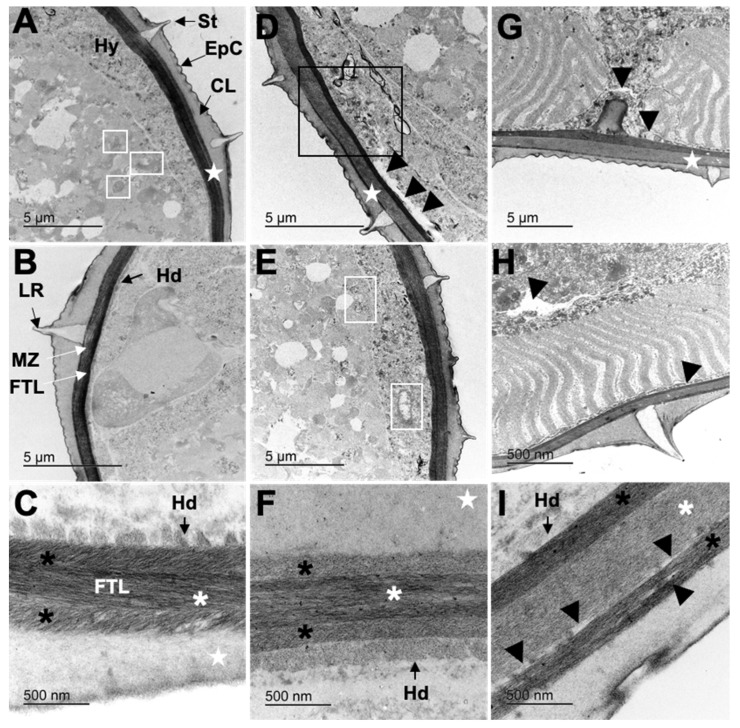
Transversal cut of adult worms of *C*. *punctata* observed through Transmission Electron Microscopy. (**A**–**C**) Transversal cut of adult worms of *C*. *punctata* from the control group (ethanol 2.5%); showing the epicuticle (EpC), pseudometric annulations (cuticular grooves), cortical layer (CL), Strout (St) forming the longitudinal ridges (LR), hypodermis (Hy), medial zone (MZ), the fibrous tri-layer from the basal zone (FTL), and the hemidesmosomes (Hd); (**D**–**F**) transversal cut of adult worms of *C*. *punctata* incubated in CuQ where tumefaction of mitochondrion can be observed (white square) and an alteration of the fibrous tri-layer of the BZ; (**G**–**I**) transversal cut of adult worms of *C*. *punctata* incubated in CaR with hypodermic detachement and alterations of the fibrous tri-layer of the BZ.

In the basal zone (BZ) of all treated L_3_, the collagen fibers showed an increased electron lucency and lower definition of the vertical stripes pattern of collagen fibers (Figure 5C,D; white asterisks). Alterations in the electron density of the seath’s internal layer (Figure 6B–D; black star), hemidesmosomes (Hd) (Figure 5B–D, Figure 6C, Figure 7C and Figure 8C), dense bodies (DB) (Figure 6B,D) and union plates (UP) were also observed (Figure 6C).

The somatic cell muscle (ScM) of larvae exposed to CaR displayed a myofilament disorganization on the A band of some sarcomeres with an apparent reduction in the number of TnM (Figure 6C, oval shape) due to an increased electron lucency of TnM (white asterisk) and increased electron density of TkM (black asterisk). The alterations previously described were also observed for the positive control, although in the TBZ treatment, L_3_ showed a stronger degeneration of TkM (Figure 6D, black asterisk).

Finally, micrographs of the lateral end of *Cooperia punctata* L_3_ allowed the visualization of the cuticle–sheath complex and epithelial specializations (Figure 7A–D). Up to 100% of specimens exposed to the treatments lost the ILSp transverse band pattern and the cilia of the deirid-like neuron tip (Dn) and deirid-like neuron base (Figure 7B–D; black star).

The seam cell (SC) of treated L_3_ was also affected within all treatments, and the main lesions observed were: loss of nuclear envelope (Figure 7C,D; NcE); and vacuolization of the cytoplasm in 93.33%, 53% and 100% of L_3_ incubated in CuQ, CaR and TBZ, respectively (Va; Figure 7B–D). Condensation and margination of the heterochromatin within the nucleus (white asterisk) was registered in 33.3% of the L_3_ incubated in CuQ and 100% of the L_3_ incubated CaR and TBZ. Finally, electron densification of the nucleus cytoplasm was evident in 66.67% and 7% of L_3_ exposed to CuQ and CaR, respectively.

#### 3.4.2. Adult Worms

Figure 8A–C present a transversal section of adult worms of *C*. *punctata* from the control group (ethanol 2.5%) showing the cuticle (white star), and the cortical layer (CL) from which emerges the strout (St) to form the longitudinal ridges (LR) (Figure 8B). The hypodermis (Hy) presents a homogeneous electron density and several mitochondria (Figure 8A, white squares). In the cuticle, we can distinguish the medial zone (MZ), the fibrous tri-layer from the basal zone (FTL), and the hemidesmosomes projected from the basal zone and connected with epithelial cells (Hd; Figure 8C). Figure 8C allows visualization of the FTL present in the basal zone with two well-defined external layers in a spiral disposition (black asterisks) that are crossed by a third layer of fibers that are longitudinally disposed (white asterisk).

Parasites incubated in PC combinations showed a slight electron densification of the CL, however, an increased electron lucency in the MZ was evident in all treated worms. Furthermore, parasites exposed to CuQ combination presented higher electron lucency of the three-layer fibers from the basal zone with a marked loss in the fiber’s definition, apparent length reduction, and an increased angle of the spiral fibers with respect to the longitudinal layer (Figure 8F; black asterisk and white asterisk, respectively). Hemidesmosomes (Hd) showed a reduced electron lucency and definition (Figure 8F), and the hypodermis reveals cell derangement and electron densification (Figure 8D; black square) with electron-lucent zones suggesting hypodermic detachment (Figure 8D; black triangles). Moreover, 75% of parasites exposed to CuQ combination presented mitochondrion swelling and electron density alterations in both mitochondrial cristae and matrix (Figure 8E; white squares).

Lastly, in all analyzed *C. punctata* worms incubated in the CaR combination, the FTL had a significant increase in electron density in both spiral layers and an increased electron lucency of the longitudinal layer (Figure 8I; black asterisk and white asterisk, respectively). Electron-lucent spaces through the layers were also observed (Figure 8I, black triangles) suggesting detachment between fibrous layers. Finally, an apparent shortening of the fibers length that could be associated to an increase in the spiral angle disposition with respect to the longitudinal fibrous layer.

## 4. Discussion

### 4.1. Motility Inhibition of Polyphenolic Compounds Combinations (PCs-Combinations)

Development of new drugs is a time consuming and expensive industry, which can take up to 15 years, cost around $1.2 billion dollars and has only a 5% success rate [6]. Even though most drug-engineering technologies have rapidly improved over the years, there has been an anthelmintic drug-underdevelopment, which could be attributed to the poor knowledge of parasite biology [6]. In vitro assays as a tool for new drug screening is more expensive and time consuming; however, it allows us to target different biological stages of the most important parasites [6]. The molecules and combinations used in this study were selected from previous research reporting their AH-like activity against egg hatching and larval exsheathment of *C*. *punctata* [7]. In accordance with Paolini et al. (2003) [14], the results of this research showed that the concentration of PCs used have a higher activity against adult motility than ensheathed L_3_; which could be associated with the differences in molecular binding sites of both biological stages of the nematode. Multiple studies have reported that the susceptibility of GIN to organic compounds with AH-like activity could be directly related to the parasitic genera, life stage and even their site of establishment within the host [13,14,15,16,17]. Furthermore, authors suggest that the cuticles of parasitic stages are more susceptible to anthelmintic compounds and affect the motility of adult worms; opposite to free-living stages, to whom their specialized structures provide protection towards environmental distress [15,18].

The screening for anthelmintic-like activity of plant secondary metabolites against adult nematodes has been mainly performed via assessing tannin-rich plant extracts [11,19]. Authors have reported total paralysis of *C. oncophora* at ≥ 500 μg extract mL^1^ [11], results which are very similar to the EC_50_ obtained with both combinations used in this study, CuQ and CaR: 0.073 ± 0.071 mg mL^−1^ and 0.051 ± 0.164 mg mL^−1^, respectively. However, unlike Peña-Espinoza et al. (2017) [11], the EC_50_´s were obtained after 24 h when the motility of the control group was still at 74.54 ± 6.17%. Observations performed after 48 h revealed a total death of the worms from the control group, which could be associated with the non-use of RPMI media as suggested by other authors [11,20].

### 4.2. Structural and Ultrastructural Alterations Observed through SEM and TEM in Both L_3_ and Adult Worms of C. punctata

In this study, the main alterations observed through SEM and TEM for both L_3_ and adult worms were: (i) alterations of the sheath–cuticle complex of L_3_, with loss of epicuticle continuity, degradation of collagen bands of the basal zone and in the lateral specializations of the internal layer of the sheath; (ii) changes in hemidesmosomes; (iii) lateral hypodermic cord deirid-like neuron structure alterations; (iv) seam cell apoptosis; (v) mitochondrial swelling; (vi) electron densification, change of angle and length of the helicoidal fibers from the basal zone of adult worms; and (vii) mild and moderate degeneration of thin myofilaments in the sarcomeres of L_3_ and adult worms, respectively.

The alterations observed in the sheath–cuticle complex of L_3_ are suggestive of the protective effect of the sheath against chemical agents [21] and consistent with their reported capacity to inhibit larval exsheathment [7]. To our knowledge, this research paper is the first one to describe both the specializations present in the inner layer of the sheath and the deirid-like neuron structure observed in the cuticle of *C*. *punctata* L_3_. The deirid neuron has been widely studied in *C*. *elegans* larvae; and it has been categorized as a sensorial organ that allows the nematode to perceive environmental stimuli (oxygen levels, social feeding behavior, attraction/repulsion to chemicals, reproductive behavior, and temperature), allowing them to react toward those stimuli with actions such as molting and hypobiosis [22]. Thus, it could be suggested that the alterations observed in both the inner layer specializations and the deirid-like neuron of *C*. *punctata* L_3_ could be responsible for the exsheathment blockage generated by these PCs-combinations and reported in previous research [7]. Further research is suggested to corroborate the nature and function of both structures observed in the sheath internal layer and in the lateral hypodermic cord of *C*. *punctata* L_3_.

Additionally, the seam cell has been described in *C*. *elegans* to have the ability to both self-renew and to give rise to differentiated cell types (hypodermal, glial and neuronal cells) [23]; thus, the alterations observed in both the seam cell and mitochondria of parasites exposed to the PC combinations could be considered to not be compatible with the development and survival of both infective larvae and adult worms.

Furthermore, alterations in cuticular collagen and structural collapse observed for both L_3_ and adult worms incubated in both PC combinations, could be associated to the bioactive properties previously described for caffeic acid and coumarin, which include: (i) fibrinolytic activity and (ii) inhibition of the epidermic growth factor and metalloproteinases, which are expressed in the hypodermic tissue of nematodes and are necessary for normal collagen secretion [15,24].

Even though the anthelmintic-like mechanism of most phytochemicals is yet to be determined, it has been proposed that motility inhibition is a result of phytochemical blocking of sensory neurons, and such interference of nematodes’ neurophysiology leads to their paralysis [25]. In that regard, recent studies have reported the acetylcholinesterase (AChE) inhibitory activity of polyphenolic compounds such as caffeic acid, coumarin and quercetin [2]. The enzyme that is essential for the regulation of cholinergic transmission in nematodes [26] and target for drug-engineering; Levamisole being one of the most known examples of cholinergic agonist drugs affecting the neuromuscular synapses of nematodes [27,28]. Caffeic acid, coumarin and its derivatives have been reported to be phytotoxins with a neurotoxic effect inhibiting the AChE activity in neuromuscular junctions [28,29]. Furthermore, coumarin has also been reported to block the octopamine receptor pathway [28]. Octopamine is a biogenic amine, which among others, is involved in the modulation of processes of pharyngeal pumping, muscle contractions and oviposition of female nematodes [30]. It is feasible that, one or the combination of both neurotoxic effects of coumarin and caffeic acid previously described are directly associated with the anthelmintic-like activity observed against adult worms of *C*. *punctata*; thus, further studies are required to corroborate this hypothesis.

However, mechanical alterations should also be addressed, as nematodes, as with most of the cylindrical-shaped animals, have a hydrostatic skeleton; in which movement depends on the internal fluids, the longitudinal muscle fibers and in the crossed-fiber helical array of connective tissue present in the basal zone of the cuticle [31,32]. It has been reported that the disposition of the different muscle fibers combined with the tri-layer connective tissue fibers (stiff and inextensible), and the pressurized internal fluid of nematodes allow cuticle deformation, therefore enabling locomotion. Thus, the coupling between body wall muscles and the cuticle implies that muscle contraction could dynamically affect the morphology of the nematode’s cuticle [33]. Moreover, the cuticular ridge pattern (both transversal and longitudinal) confer the flexibility features of the cuticle, allowing nematodes to coil, assist locomotion and favor the attachment of nematodes to the host intestinal mucosa [34]; while lateral alae provide longitudinal stiffness and enable the diameter shift of nematodes’ body shape [35]. Scanning and Transmission Electron Micrographs obtained through this investigation allowed the classification of specific treatment-induced lesions in both the cuticle and muscular tissue of L_3_ and adult worms of *C*. *punctata*, involving the structures previously described, which could have led to the paralysis observed.

The structural alterations observed in the specimens are consistent with previous reports that correlate cuticular collagen alterations with the cylindric shape of nematodes. As collagen and other similar proteins constitute 80% of the structural components of parasites’ cuticles [15]. Scanning Electron Micrographs of L_3_ revealed loss of turgor and collapsed morphology; lesions that might be associated to the loss of cuticular ridges pattern and of the striated collagen layer observed in the cuticle basal zone; structures which according to Wharton (1986) confer nematodes with progressive resistance to deformation. Furthermore, hemidesmosomes alterations in both biological stages could also be involved in the structural changes observed in the nematode’s morphology; as hemidesmosomes are trans-cellular protein complexes that promote the epidermal adherence, helping to maintain the tissue structure and integrity by providing resistance to mechanical stress [36]. Thus, changes in hemidesmosomes electron density could also be associated with the collagen degradation capacity of the PC combinations, and motif for the presence of electron-lucent zones suggesting hypodermic detachment with the cuticle.

Furthermore, and consistent with the lesions observed through SEM, Petzold (2011) [33] reported that treatments inducing rigid paralysis, such as ACHs inhibitors, provoke the head and tail region to collapse more frequently than the midbody of nematodes; while the changes regarding body length, diameter, and body stiffness are representative of alterations of the body wall muscle tone.

Moreover, TEM microphotographs of specimens treated with caffeic acid evidenced an apparent loss of thin myofilaments and the electro-densification of thick myofilaments in both L_3_ and adult worms’ muscular cells. Findings consistent with previous studies reporting that caffeic acid, rutin and quercetin affect myofibrillar proteins of muscular tissue [37]. Cheng et al. (2020) [37] reported caffeic acid as the polyphenolic compound that affects muscular tissue the most via inhibiting collagen production, decreasing the alpha-helix structure of thin myofilaments and increasing beta-sheets and beta-turns of thick myofilaments, alterations that trigger coiling of muscular tissue. The changes observed through TEM in *C*. *punctata* myofilaments and in the crossed-fiber helical connective tissue of the basal zone are consistent with previous reports affirming that muscle contraction shortens the body shape and increases the body stiffness of nematodes [33], showing a consistency in the lesions observed through both SEM and TEM techniques.

Moreover, it has been reported that the shortening of the nematodes’ body shape compels an increase in diameter, which is resisted by the crossed-fiber helical connective tissue array [31]. Such alterations could be directly associated with the turgor loss in the case of L_3_, and in the case of adult worms, it could be related to the apparent thin myofilament degeneration within the sarcomere and to the reduction in length and angle disposition of the tri-layered helicoidal fibers from the basal zone of the cuticle. Concluding that, the possible mechanism of action in adult worms is a rigid paralysis and muscular tone alterations.

Lastly, apoptotic cells observed through TEM micrographs with both treatments are consistent with previous reports describing caffeic acid as a time and concentration dependent proapoptotic molecule, which promotes chromatin condensation [38] and induces mitochondria-mediated apoptosis [39]. Similarly, coumarin has been reported with anti-leishmania activity causing both cytoplasm vacuolization and mitochondrial swelling [40]; lesions that were observed in this study after incubation of both L_3_ and adult worms in both PC combinations.

Combination of in vitro bioassays and Electron Microscopy techniques allowed us to conclude that although some enzymatic inhibition could impair *C*. *punctata* viability, mechanical alterations are also involved in the PC combinations bioactivity and are conclusive for the effectiveness of both PC combinations to affect collagen and muscular tissue of *C*. *punctata* adult worms causing muscular contraction, leading to cuticular collapse, loss of turgor pressure and cellular damage. The motility of infective larvae was less affected, most likely because of the resistance conferred by the sheath; however, mild damage was observed in the muscle tissue. Nevertheless, the sheath, cuticle and cellular alterations are consistent with the exsheathment impairment of both PC combinations that was previously reported by Escareño-Díaz et al. (2019) [7]. Further studies assessing the effect of PC combinations on mortality rates and over the motility of exsheathed larvae are suggested.

## 5. Conclusions

The results of the present study led us to conclude that both PC combinations have anthelmintic-like activity against both biological stages of *C*. *punctata*; thus, motility inhibition could only be stated against adult worms. After addressing their potential toxicity and pharmacokinetics, both combinations could be considered for in vivo field trials.

## Figures and Tables

**Table 1:** *Cooperia punctata* larval motility inhibition after exposure to polyphenols combinations.

Treatment	Concentration (mg mL^−1^)	Motility Inhibition% (Mean ± SE)
Thiabendazole	10	88.52 ± 6.59 ^a^
Coumarin: quercetin	0.8	3.82 ± 1.87 ^b^
Caffeic acid: Rutin	0.84	13.95 ± 4.84 ^c^

Different letters within a column represent statistically significant differences among treatments.

**Table 2:** Mean effective concentrations (CE_50_), confidence intervals 95% (IC) and correlation coefficients (R^2^) of the combination of polyphenolic compounds against *Cooperia punctata* adult motility.

Combination	Time Lapse (h)		EE	Limits IC 95%		R^2^
		EC_50_ (mg mL^−1^)		Inferior	Superior	
Coumarin: Quercetin	6	1.621	0.154	0.944	12.41	0.87
	12	0.398	0.064	0.267	0.677	0.93
	24	0.073	0.071	0.045	0.117	0.94
Caffeic acid: Rutin	6	NE	NE	NE	NE	NE
	12	0.192	0.061	0.129	0.288	0.95
	24	0.051	0.164	0.0008	0.125	0.75

NE: Could not be estimated.

## Data Availability

No new data were created or analyzed in this study. Data sharing is not applicable to this article.
